# Temporal trends and characteristics of fall-related deaths, hospitalizations and emergency department visits among older adults in Canada

**DOI:** 10.24095/hpcdp.44.11/12.04

**Published:** 2024-11

**Authors:** Xiaoquan Yao, Andr S. Champagne, Steven R. McFaull, Wendy Thompson

**Affiliations:** 1 Behaviour, Environment, and Lifespan Division, Centre for Surveillance and Applied Research, Health Promotion and Chronic Disease Prevention Branch, Public Health Agency of Canada, Ottawa, Ontario, Canada

**Keywords:** accidental falls, aged, death, mortality, hospitalization, emergency room visits, men, women

## Abstract

Falls among older adults (aged 65 years and older) are a public health concern in Canada. Fall-related injuries can cause a reduction in quality of life among older adults, and death. They also entail substantial health care costs. It is essential to monitor fall-related injuries and deaths among older adults to better understand temporal trends and characteristics and to evaluate fall prevention strategies. We used the most up-to-date data from the Canadian Vital Statistics–Death database, Discharge Abstract Database and National Ambulatory Care Reporting System to analyze the temporal trends of fall-related mortality, hospitalizations and emergency department (ED) visits among older adults in Canada over more than a decade. Age and sex characteristics were also examined. In 2022, 7189 older adults died due to a fall in Canada (excluding Yukon). From 2010 to 2022, deaths due to falls generally increased in both number and rates. In fiscal year 2023/24, there were 81 599 fall-related hospitalizations in Canada (excluding Quebec) and 212 570 fall-related ED visits in Ontario and Alberta. From fiscal year 2010/11 to 2023/24, even though the overall trend of the rates of fall-related hospitalizations and ED visits did not increase, the numbers generally rose year by year except in 2020/21, the early stage of the COVID-19 pandemic. As for the age and sex characteristics, the rates for deaths, hospitalizations and ED visits rose with advancing age for both men and women. With the aging population, continuous monitoring of the trends is crucial for fall prevention.

**Highlights**

- In 2022, the number of deaths due to falls among older adults was 7189, representing a crude rate of 98.2 per 100 000 older adults in Canada (excluding Yukon). The age-standardized rate increased annually at 1.7% from 2010 to 2013, followed by an annual decrease of 2.8% from 2013 to 2016, and increased again annually at 6.8% from 2017 to 2019 and 4.1% from 2019 to 2022.
- In fiscal year 2023/24, the number of fall-related hospitalizations among older adults was 81 599, representing a crude rate of 14.1 per 1000 older adults in Canada (Quebec excluded). The age-standardized rate increased annually at 1.4% from 2010/11 to 2013/14 and decreased annually at 0.4% from 2013/14 to 2019/20. The 2020/21 rate showed a decrease (7.0%) compared to the pre-COVID-19 year 2019/20. After 2020/21, the age-standardized rate increased, 4.0% higher in 2021/22 compared to 2020/21, and 2.1% higher in 2023/24 compared to 2022/23.
- In fiscal year 2023/24, the number of fall-related ED visits among older adults was 212 570, representing a crude rate of 58.9 per 1000 older adults in Ontario and Alberta combined. The age-standardized rate increased annually at 1.6% from 2010/11 to 2017/18. The 2020/21 rate showed a sharp decrease (16.8%) compared to the pre-COVID-19 year 2019/20. After 2020/21, the age-standardized rate greatly increased in 2021/22, 13.7% higher compared to 2020/21. The 2022/23 rate was 1.0% higher than that of 2021/22 and the 2023/24 rate was 2.1% higher than that of 2022/23.

## Introduction

Falls among older adults (aged 65 years and older) are a public health concern worldwide. According to the WHO, about a third of older adults fall each year.^1^ In Canada, 5581 older adults died due to falls in 2019, representing a crude mortality rate of 84.6 per 100 000 older adults. The age-standardized mortality rate increased by 111% from 2001 to 2019. Between fiscal years 2008/09 and 2019/20, the annual number of fall-related hospitalizations (FRHs) among older adults increased by 47% from 49 152 to 72 392 (Quebec data not available). However, the age-standardized FRH rate was relatively stable during this time period, at approximately 15 per 1000 older adults. FRHs represented 87% of all injury-related hospitalizations among older adults in Canada (excluding Quebec).^2^

Falls and their resulting injuries cause loss of life, reduce quality of life and entail substantial health care costs. In 2018, the annual direct cost of injurious falls among older Canadian adults was estimated at CAD 5.6 billion, which was more than twice the cost associated with falls among those aged 25 to 64 years.^3^

Older adults are projected to represent over a fifth of the Canadian population by 2068.^4^ Therefore, it is essential to monitor trends in falls among this population and the associated burden on those injured, their families and the health care system, which is important for effective management and prevention. The objective of this study was to use the most recent data available to provide the temporal trends and characteristics of fall-related mortality, hospitalizations and emergency department (ED) visits among older adults in Canada.

## Methods

Our data sources were the Canadian Vital Statistics–Death database^5^ for deaths due to falls, the Discharge Abstract Database (DAD)^6^ for FRHs and the National Ambulatory Care Reporting System (NACRS)^7^ for fall-related ED visits.

We used ICD-10/ICD-10-CA^8^,^9^ codes W00–W19 (unintentional fall) to identify fall cases. For deaths due to falls, we used the underlying cause, that is, the disease or external cause of injury that initiated the sequence of events leading directly to death, or the circumstances of the incident that produced the fatal injury. A fall-related hospitalization is defined as a hospitalization in acute care containing W00–W19 in the diagnosis fields (maximum of 25) in the DAD record.^2^,^10^ The analysis was based on episodes of care. If a patient was transferred for care to another health facility, all discharges were counted as a single case (or episode).^2^,^11^,^12^ We merged DAD records with an exact match on (1) encrypted health card number; (2) health card issuing province; and (3) year of birth.^2^,^11^,^12^ Fall-related ED visits were identified by any one of the diagnoses (maximum of 10) containing W00–W19 in the NACRS ED records.^2^ A similar analysis based on episodes of care and linking methodology was also conducted for ED visits.

We used SAS Enterprise Guide version 7.1^13^ to compile the pooled and stratified (by sex and age) counts. Population estimates from Statistics Canada^14^ were used for rate calculation. We used the 2011 Canadian population for direct age standardization. To quantify temporal trends, we used Joinpoint software version 5.0.2^15^ to compile annual percent changes (APCs) of age-standardized rates. Considering the interruption in hospitalizations and ED visits caused by the COVID-19 pandemic, as of fiscal year 2020/21, APC was calculated by the difference in hospitalization or ED visit rates between two continuous years divided by the first year. We checked the autocorrelation in the data and used the corresponding setting in Joinpoint to run the program. A *p* value threshold of 0.05 was used to determine statistical significance.

## Results

***Temporal trends in deaths, hospitalizations and ED visits***

**Figure 1:**
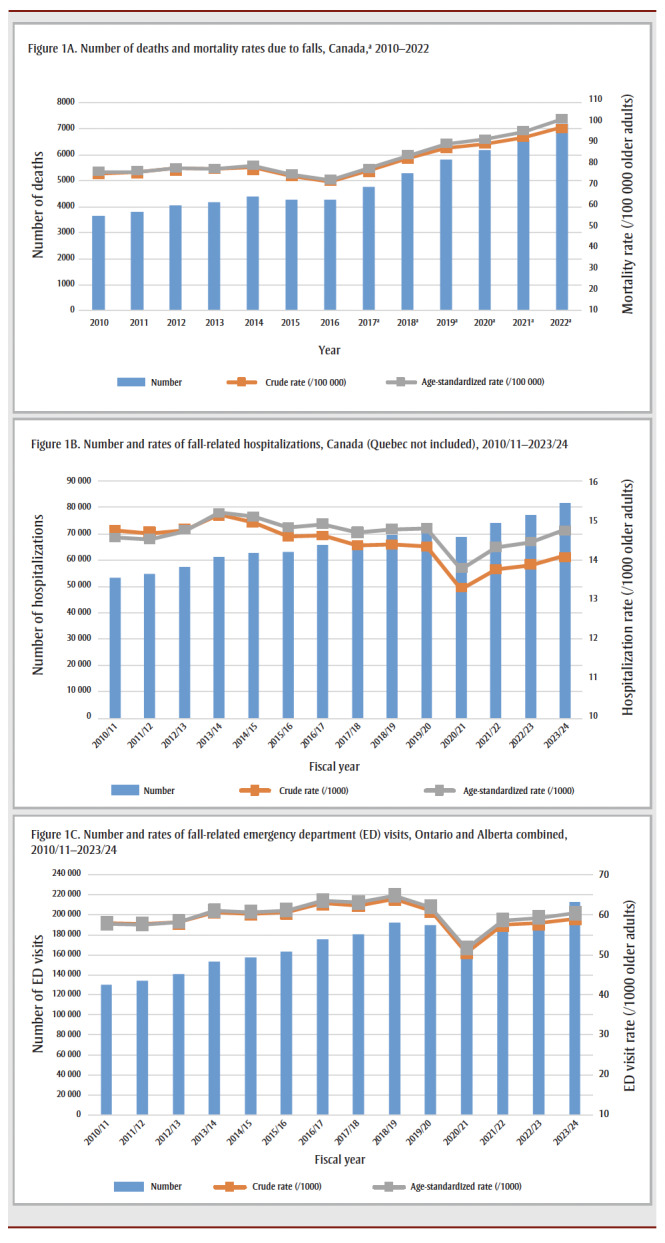

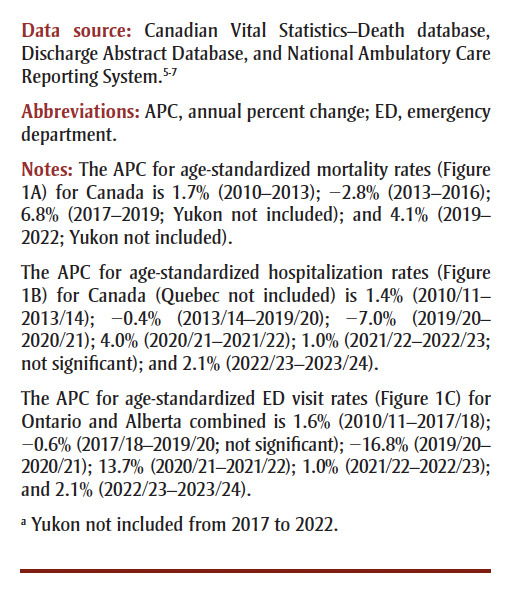
Temporal trends of fall-related deaths, hospitalizations and emergency department visits among older adults in Canada

Figure 1 shows the temporal trends in deaths due to falls, and fall-related hospitalizations and ED visits over more than a decade. Annual numbers and crude and standardized rates are presented.

In Canada (all provinces and territories), the annual number of deaths due to falls rose between 2010 (3652) and 2014 (4383) with decreases in 2015 (4274) and 2016 (4252). From 2017 to 2022, in Canada (excluding Yukon), the annual number of deaths due to falls rose more steeply, reaching 7189 in 2022 (crude rate: 98.2/100 000 older adults). The age-standardized rate increased annually at 1.7% from 2010 to 2013, decreased annually at 2.8% from 2013 to 2016, and increased again annually at 6.8% from 2017 to 2019 and 4.1% from 2019 to 2022.

For FRHs (excluding Quebec), the annual numbers steadily rose from fiscal year 2010/11 (53 347) to 2023/24 (81 599) except in 2020/21 (68 759), the year of the early stage of the COVID-19 pandemic. The number of hospitalizations in 2023/24 was 53.0% higher than in 2010/11. The trend in crude rates for hospitalizations showed different results and was highest in 2013/14 (15.1/1000 older adults); the rate was 14.1 per 1000 older adults in 2023/24. The age-standardized rate increased annually at 1.4% from 2010/11 to 2013/14 and decreased annually at 0.4% from 2013/14 to 2019/20. The 2020/21 rate showed a decrease (7.0%) compared to the pre-COVID-19 year 2019/20. After 2020/21, the age-standardized rate increased: 4.0% higher in 2021/22 compared to 2020/21, 1.0% higher in 2022/23 compared to 2021/22 (not significant) and 2.1% higher in 2023/24 compared to 2022/23.

In Ontario and Alberta, two provinces where full data coverage is available, the annual number of fall-related ED visits continuously rose from fiscal year 2010/11 (129 825) to 2018/19 (191 689) with a slight decrease in 2019/20 (189 669). The number greatly decreased in 2020/21 (163 026) but rose again, above all the previous years, with 212 570 visits in 2023/24. The crude rate was 58.9 per 1000 older adults in 2023/24 and highest in 2018/19 (64.0/1000 older adults). The age-standardized rate increased annually at 1.6% from 2010/11 to 2017/18. The 2020/21 rate showed a sharp decrease (16.8%) compared to the pre-COVID-19 2019/20. After 2020/21, the age-standardized rate greatly increased in 2021/22, 13.7% higher compared to 2020/21. The 2022/23 rate was 1.0% higher than that of 2021/22 and the 2023/24 rate was 2.1% higher than that of 2022/23.

***Age and sex characteristics***

**Figure 2:**
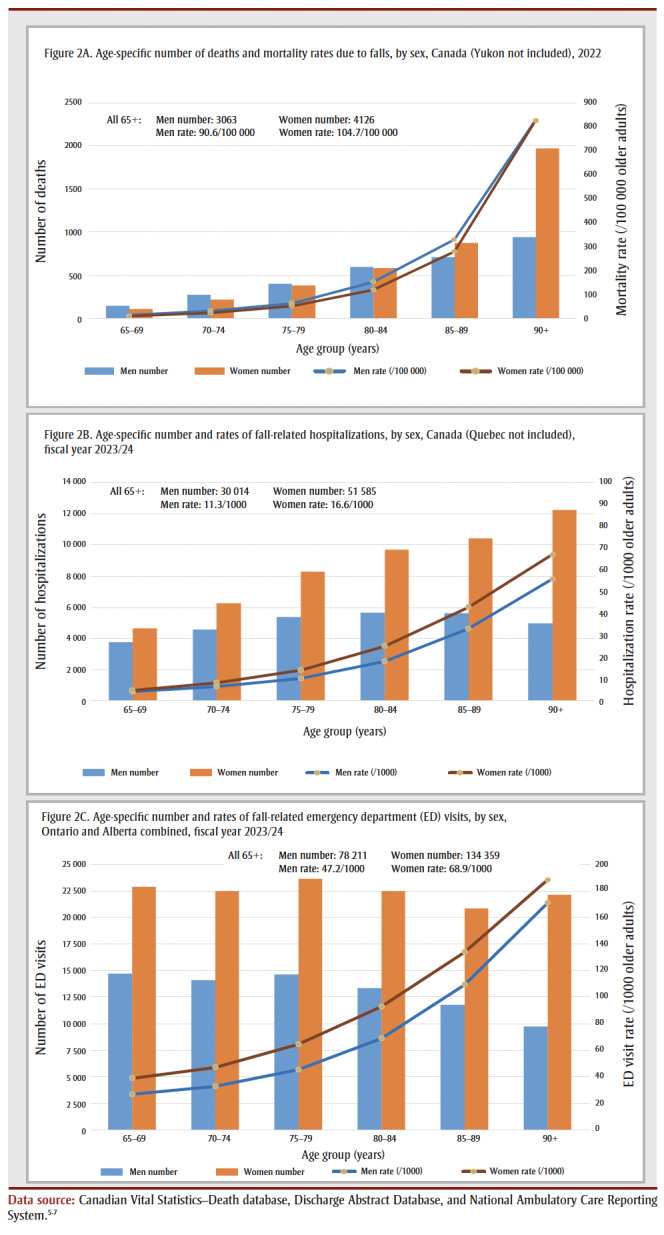
Age-specific number and rates of fall-related deaths, hospitalizations and emergency department visits, by sex, among older adults in Canada

Figure 2 shows the age-specific number and rates by sex for 2022 for deaths and fiscal year 2023/24 for fall-related hospitalizations and ED visits.

In 2022, 7189 older adults (3063 men and 4126 women) died due to a fall in Canada (excluding Yukon). The age-specific mortality rates rose with advancing age for both men and women. Men had higher rates than women across all age groups even though more women than men died from falls.

In fiscal year 2023/24, there were 81 599 FRHs (excluding Quebec) among older adults, almost two-thirds of which (51 585) occurred among women. The age-specific rates were higher at successively older ages, and numbers and rates for FRHs in women consistently exceeded those of men.

In fiscal year 2023/24, there were 212 570 fall-related ED visits in Ontario and Alberta among older adults, almost two-thirds of which (134 359) occurred among women. Similar to FRHs, the age-specific rates were higher at successively older ages, and the numbers and rates for women consistently exceeded those of men.

## Discussion

We used the most up-to-date data to analyze the temporal trends and characteristics of deaths due to falls, and fall-related hospitalizations and ED visits for older adults in Canada, spanning over a decade. From 2010 to 2022, deaths due to falls for those aged 65 years and over generally increased in both numbers and rates. From fiscal year 2010/11 to 2023/24, even though the overall trend of the rates of fall-related hospitalizations and ED visits did not increase, the numbers generally rose year by year, except in 2020/21, the early stage of the COVID-19 pandemic. As for age and sex characteristics, the rates for deaths, hospitalizations and ED visits rose with advancing age for both men and women.

We observed a difference between the trends for mortality and morbidity. The overall trend of the rates of fall-related hospitalizations and ED visits has been relatively steady (except in 2020/21), with the numbers increasing. This indicates that the overall risk of going to the ED or being hospitalized because of a fall remains relatively stable, and that the rise in numbers is probably mostly due to the aging population. However, the mortality due to falls has increased in both rates and numbers. Further research is needed to explain this, such as the trend in the nature of injuries resulting from falls, and comorbidities.

The results of this paper demonstrate that falls among older adults remain an important public health concern in Canada despite prevention efforts. Risks associated with falls stem from a number of biological, behavioural, socioeconomic and environmental factors.^16^ Older adults have a particularly high risk of falling compared to the younger population. This risk can be attributed to a number of factors, which can include decreased mobility and balance, muscle weakness, visual impairment and medication side effects.^16^

Fall prevention efforts generally entail a multifaceted approach. At the individual level, for instance, participating in balance and strength exercises and managing medications and their side effects are strategies that may reduce the risk of falls among older adults. At the community level, providing educational opportunities to older adults on fall prevention strategies (i.e. exercise programs, fall prevention skills and social connection), installing handrails and grab bars, and removing hazards such as snow and ice from public walkways are examples of broader-scale approaches.^17^,^18^ Continued research to address knowledge gaps and assess promising practices will strengthen the evidence base, lessen the consequences of falls for older adults and promote healthy aging.

***Strengths and limitations***

The strengths of this study include the timeliness of data, the analysis of both mortality and morbidity related to falls among older adults and the quantification of the temporal trends over a decade.

Our results are subject to the limitations existing in the data sources we used. The Canadian Vital Statistics–Death database does not include information from Yukon as of 2017; the mortality counts in years 2019 to 2022 are to be considered preliminary. With respect to hospitalizations, Quebec data were not included. Additionally, data for ED visits came from Ontario and Alberta only. All of these limitations pose a difficulty in presenting a complete national picture in Canada. 

## Conclusion

The health care burden of falls (deaths due to falls, fall-related hospitalizations and ED visits) among older adults in Canada increased from 2010 to 2022. The information presented in this paper is essential for understanding the temporal trends in falls and patient characteristics and evaluating fall prevention strategies among older adults in Canada. As Canada’s population ages, continuous monitoring will be crucial.

## Conflicts of interest

All authors declare no conflicts of interest.

## Authors’ contributions and statement

XY: conceptualization, formal analysis, methodology, writing—original draft.

AC: writing—review and editing.

SM: writing—review and editing.

WT: writing—review and editing, supervision.

The content and views expressed in this article are those of the authors and do not necessarily reflect those of the Government of Canada.
